# Colonoscopy-detected high-risk adenomas and their association with alcohol consumption among first-degree relatives: an observational case–control study

**DOI:** 10.3389/fonc.2026.1791247

**Published:** 2026-05-21

**Authors:** Yuan Lin, Hailong Zhu, Dan Zhang, Jinbi Xie, Mengjing Pan, Shanjuan Wang

**Affiliations:** 1 Department of Gastroenterology, Jiading District Central Hospital Affiliated Shanghai University of Medicine & Health Sciences, Shanghai, China; 2 Department of Medical Oncology, Minhang Branch, Fudan University Shanghai Cancer Center, Shanghai, China

**Keywords:** alcohol consumption, colonoscopy, colorectal adenoma, first-degree relatives, high-risk adenoma, predictive model, associated factors, risk stratification

## Abstract

**Background:**

First-degree relatives (FDRs) of individuals with colorectal neoplasia are at increased risk of advanced colorectal lesions. However, risk stratification within this high-risk population remains suboptimal. This study aimed to identify factors associated with high-risk adenomas (HRAs) among FDRs and to evaluate the predictive performance and clinical utility of a multivariable model integrating lifestyle, clinical, and laboratory variables.

**Methods:**

In this observational case–control study, 210 FDRs undergoing colonoscopy were enrolled, including 105 FDRs of probands with HRAs and 105 FDRs of probands with normal colonoscopy findings. Baseline characteristics, lifestyle factors, fecal immunochemical test (FIT) results, and serum biomarkers were collected. Univariable and multivariable logistic regression analyses were performed to identify factors associated with HRAs. Model performance was assessed using ROC curves, calibration analysis, and decision curve analysis (DCA), with internal validation using bootstrap resampling; however, no external validation was performed.

**Results:**

HRAs were detected in 34.8% of FDRs in this study sample. Alcohol consumption was significantly associated with higher odds of HRAs in both univariable (OR = 4.30, 95% CI: 1.54–11.98, P = 0.005) and fully adjusted models (OR = 5.75, 95% CI: 1.49–22.20, P = 0.011). In contrast, proband HRA status was not significantly associated with HRAs among FDRs. The prediction model demonstrated moderate discrimination (area under the curve = 0.707) and acceptable calibration, with potential clinical utility primarily at low-to-intermediate threshold probabilities, although net benefit became less stable at higher thresholds. The observed difference in the proportion of FDRs with HRAs between groups was smaller than expected.

**Conclusion:**

A substantial proportion of FDRs had HRAs detected, underscoring the importance of targeted screening in this population. Alcohol consumption appears to be an important modifiable factor associated with HRAs, whereas proband HRA status provides limited additional value for risk stratification. The proposed model may aid in risk stratification, although further studies incorporating genetic and environmental factors and external validation are needed to improve predictive accuracy. These findings support the need for colonoscopy-based screening and targeted lifestyle interventions among FDRs.

## Introduction

Colorectal cancer (CRC) remains a major global public health burden and a leading cause of cancer-related morbidity and mortality worldwide (1). The majority of CRCs develop through a well-established adenoma–carcinoma sequence, highlighting the critical importance of early detection and removal of precursor lesions, particularly advanced or high-risk adenomas (HRAs), for effective cancer prevention (2, 3). Colonoscopy-based screening with polypectomy has been shown to substantially reduce CRC incidence and mortality, supporting strategies focused on identifying individuals at increased risk of advanced neoplasia (2, 3).

Current screening guidelines recommend risk-stratified approaches to optimize screening modalities and timing (4, 5). Individuals with a positive family history, especially first-degree relatives (FDRs) of patients with colorectal neoplasia, are consistently recognized as a higher-risk group (4, 6). Previous studies suggest that FDRs of individuals with advanced adenomas also carry an elevated risk of clinically significant colorectal lesions, even in the absence of overt CRC in the proband (7–9). However, risk stratification within this already high-risk population remains insufficiently refined, particularly in identifying which individuals are at highest risk of advanced lesions beyond family history alone. Importantly, it remains unclear whether the presence of HRAs in probands provides additional discriminatory value for risk stratification among their FDRs beyond shared familial and environmental factors.

In addition to family history, other modifiable and clinically accessible factors may contribute to improved risk stratification among FDRs. Alcohol consumption has been suggested as a potential modifiable risk factor for colorectal neoplasia, with evidence supporting its role in adenoma development. However, its association with advanced adenomas, particularly among high-risk populations such as FDRs, remains incompletely characterized. Commonly used biomarkers, such as carcinoembryonic antigen (CEA), carbohydrate antigen 19-9 (CA19-9), and carbohydrate antigen 72-4 (CA72-4), as well as fecal immunochemical testing (FIT), have been widely applied in colorectal cancer screening or surveillance; however, their utility in identifying premalignant lesions remains uncertain, particularly given the limited sensitivity of FIT for advanced adenomas (10–12).

Given these considerations, there is a need to more effectively integrate clinical, lifestyle, and laboratory factors to improve risk stratification among FDRs. Moreover, beyond identifying associations, evaluating the predictive performance and clinical utility of such factors is essential for translating findings into practical screening strategies.

Therefore, in this observational case–control study, we aimed to identify factors associated with HRAs among FDRs, with a particular focus on evaluating the role of alcohol consumption and the incremental value of proband HRA status in improving risk stratification beyond family history alone. Specifically, we sought to clarify the role of alcohol consumption, evaluate the contribution of clinical and laboratory factors, and assess the performance and clinical utility of multivariable prediction models.

## Methods

### Study design and population

This frequency-matched observational case–control study was conducted at the Endoscopy Center of Shanghai Jiading District Central Hospital. The study period for first-degree relative (FDR) recruitment was from October 2023 to December 2025, while probands were identified from colonoscopy records between 2018 and 2025. Given the retrospective identification of probands and enrollment of FDRs, potential temporal differences in proband status were considered but were unlikely to substantially affect classification based on colonoscopic findings.

First-degree relatives were defined as biological parents, children, or full siblings of probands. Probands were categorized into two groups based on colonoscopic and histopathological findings: those with HRAs and those with no colorectal lesions.

### Proband-defined recruitment groups and outcome definition

First-degree relatives were recruited according to the colonoscopic and histopathological status of their corresponding probands. One recruitment group consisted of FDRs of probands diagnosed with HRAs, and the other recruitment group consisted of FDRs of probands with normal colonoscopy findings, defined as the absence of polyps, adenomas, or other colorectal lesions. A total of 105 eligible FDRs were enrolled in each proband-defined recruitment group.

FDRs of probands with normal colonoscopy findings were frequency-matched to FDRs of probands with HRAs at a 1:1 ratio by age (±5 years) and sex. Matching was performed at the group level rather than by individual pairing to ensure comparable distributions of these variables between the two proband-defined recruitment groups.

For association analyses, the outcome was defined according to the FDRs’ own colonoscopic and histopathological findings, rather than proband HRA status. Accordingly, FDRs were classified as having HRA or not having HRA for descriptive comparisons and logistic regression analyses.

### Eligibility criteria

First-degree relatives (FDRs) aged ≥18 years who were willing to undergo colonoscopy and provide blood samples were eligible for inclusion. All participants provided written informed consent prior to enrollment.

Participants were excluded if they had a personal history of colorectal cancer or inflammatory bowel disease, a colorectal cancer history across three generations, contraindications to colonoscopy, pregnancy or lactation, or severe comorbidities that could compromise the safety of colonoscopy.

To reduce clustering and ensure independence, only one FDR per proband/nuclear family was retained for analysis whenever multiple eligible FDRs were available.

### Sample size calculation

Sample size was estimated based on an expected adenoma detection rate of approximately 30% among FDRs of probands without HRAs and 50% among those with HRAs, according to previous studies (8, 13, 14). Assuming a two-sided α of 0.05 and 80% statistical power, a minimum of 93 participants per group was required to detect this difference. Considering a potential 10% non-evaluable or exclusion rate, the final target sample size was set at 105 participants per group. These assumptions were based on previously published studies evaluating adenoma detection rates among first-degree relatives. However, the observed difference in proportion of FDRs with HRAs between groups in the final dataset was smaller than initially anticipated, which may have reduced the statistical power to detect associations for variables with modest effect sizes.

### Data collection

Trained research personnel collected the baseline demographic, lifestyle, and clinical data using standardized questionnaires and physical examinations. Variables included age, sex, body mass index (BMI), smoking status, alcohol consumption, hypertension, diabetes mellitus, cardiovascular disease, and aspirin use (12, 15). Alcohol consumption was assessed as a binary variable (yes vs. no) based on self-reported history at baseline. Detailed information on alcohol quantity, frequency, and duration was not available, precluding evaluation of dose–response relationships.

### Laboratory measurements

Fasting venous blood samples were collected after an overnight fast (≥8 hours) prior to colonoscopy. Serum biomarkers were measured using standardized automated analyzers in the hospital’s central laboratory, including C-reactive protein (CRP), carcinoembryonic antigen (CEA), carbohydrate antigen 19-9 (CA19-9), and carbohydrate antigen 72-4 (CA72-4).

### Fecal immunochemical test

Fecal immunochemical testing was performed using an immunochromatographic assay. Results were initially graded according to color intensity and subsequently dichotomized as negative or positive for statistical analysis, consistent with routine clinical practice and to ensure adequate statistical power (16). This dichotomization was performed to reflect real-world clinical decision-making.

### Colonoscopy procedures and histopathology

All colonoscopies were performed using high-definition systems by experienced gastroenterologists with more than 5 years of practice. Bowel preparation quality was evaluated using the Boston Bowel Preparation Scale (BBPS), and only examinations with a total score ≥6 were included (17).

Lesions were systematically recorded in terms of number, size, morphology, and anatomical location. Histopathological evaluation was conducted by experienced gastrointestinal pathologists according to World Health Organization criteria (4, 18).

HRA was defined as the presence of any of the following: adenoma size ≥10 mm, villous histology (>25% villous component), high-grade dysplasia, or ≥3 adenomas identified during a single colonoscopy (19, 20).

### Statistical analysis

All statistical analyses were performed using R software (version 4.5.1). A two-sided *P* value < 0.05 was considered statistically significant.

### Data preprocessing

Missing data in clinical variables, except for age, sex, and colonoscopic findings, were handled using a nonparametric random forest–based method (missForest algorithm), which iteratively estimates missing values using an ensemble of decision trees and is suitable for mixed-type clinical data. The imputed dataset was used for all subsequent analyses.

### Descriptive analysis

Continuous variables were assessed for normality using the Shapiro–Wilk test and presented as mean (SD) or median (interquartile range), as appropriate.

Comparisons between groups were performed using the Student’s *t*-test or Mann–Whitney U test for continuous variables and the chi-square test or Fisher’s exact test for categorical variables.

### Univariable and multivariable logistic regression

Univariable logistic regression analyses were performed to evaluate the association between candidate variables and HRAs. Variables were selected based on clinical relevance and prior literature rather than relying solely on univariable screening, although some potential confounders (e.g., dietary factors and physical activity) were not available in the dataset. Multivariable logistic regression analyses were conducted using a stepwise adjustment approach. An unadjusted model (Model 1) was first fitted, followed by a model adjusted for demographic factors, including age, sex, body mass index, smoking status, and proband HRA status (Model 2). A fully adjusted model (Model 3) was then constructed by further incorporating clinical and laboratory variables, including aspirin use, hypertension, diabetes mellitus, cardiovascular disease, C-reactive protein (CRP), carcinoembryonic antigen (CEA), carbohydrate antigen 19-9 (CA19-9), carbohydrate antigen 72-4 (CA72-4), and fecal immunochemical test (FIT) results. Results are presented as odds ratios (ORs) with 95% confidence intervals (CIs). For CA72-4, associations with HRAs were evaluated using multivariable logistic regression models, with CA72–4 analyzed as both a continuous variable (per standard deviation [SD] increase) and as quartiles (Q1–Q4), using Q1 as the reference. Important potential confounders, such as dietary factors and physical activity, were not available in the dataset and therefore could not be included in the models, which may have introduced residual confounding.

### Bootstrap validation

To evaluate the robustness of the multivariable model, bootstrap resampling (5,000 iterations) was performed. For each resampled dataset, regression coefficients for alcohol consumption (modeled as a binary variable: yes vs. no) were estimated, and corresponding ORs were calculated. The median OR, 95% bootstrap confidence interval, and the proportions of OR >1 and P < 0.05 were reported to assess model stability.

### Model performance

Model performance was comprehensively evaluated in terms of discrimination, calibration, and clinical utility. Discrimination was assessed using receiver operating characteristic (ROC) curves and quantified by the area under the curve (AUC). Calibration was evaluated using bootstrap resampling (B = 2000) to generate calibration curves, examining the agreement between predicted and observed probabilities. Clinical utility was further evaluated using decision curve analysis (DCA) by estimating the net benefit across low-to-intermediate threshold probabilities, where the model demonstrated the most stable net benefit. Net benefit was less stable at higher threshold probabilities.

### Subgroup and interaction analyses

Subgroup analyses were performed as exploratory analyses and were interpreted with caution given the limited sample size. These analyses evaluated the consistency of associations across predefined strata, including age (<60 vs ≥60 years), proband HRA status, smoking status, hypertension, and tumor biomarkers (CEA and CA19–9 categorized by median values). Variables with sparse data were excluded from subgroup analyses to ensure model stability.

### Data availability and ethics statement

The datasets generated and analyzed during the current study are available from the corresponding author upon reasonable request, subject to ethical and privacy restrictions.

The study protocol was approved by the Medical Ethics Committee of Shanghai Jiading District Central Hospital (approval number: 2024K03) and conducted in accordance with the Declaration of Helsinki. Written informed consent was obtained from all participants prior to enrollment.

## Results

### Study population

A total of 267 first-degree relatives (FDRs) underwent colonoscopy screening. After excluding 57 participants due to incomplete colonoscopy or inadequate bowel preparation (n = 36), missing key non-imputable data, including age, sex, or colonoscopic/pathological outcome data (n = 8), and a history of colorectal cancer or other major colorectal diseases (n = 13), 210 FDRs were included in the final analysis. Of these, 105 were FDRs of probands with HRAs and 105 were FDRs of probands without colorectal lesions, corresponding to the two proband-defined recruitment groups (**Supplementary Figure 1**).

### Baseline characteristics

Baseline characteristics are summarized in **Table 1**, with participants stratified according to their own HRA status. Age and sex were broadly comparable between FDRs with and without HRAs. Alcohol consumption was significantly more frequent among individuals with HRAs, suggesting a potential positive association with HRA status (P = 0.007). In contrast, serum CA72–4 levels were lower in the HRA group (P = 0.006). However, this inverse association was not retained in multivariable analyses, suggesting that the observed difference in unadjusted comparisons may be influenced by confounding factors. Overall, most baseline characteristics were comparable between groups, indicating a relatively balanced distribution of covariates.

**Table 1:** Baseline characteristics of first-degree relatives according to their own high-risk adenoma status.

Variables	FDR without HRA (N = 137)	FDR with HRA (N = 73)	P value
Demographics			
Age, years	61.1 (12.80)	63.5 (10.50)	0.147
Male sex, n (%)	63 (46.0%)	43 (58.9%)	0.101
BMI, kg/m²	23.4 (3.60)	24.0 (3.11)	0.174
Lifestyle			
Smokers, n (%)	20 (14.6%)	15 (20.5%)	0.364
Alcohol consumption, n (%)	6 (4.4%)	12 (16.4%)	0.007
Medical history			
Proband HRA status, n (%)	65 (47.4%)	40 (54.8%)	0.385
Hypertension, n (%)	60 (43.8%)	37 (50.7%)	0.419
Diabetes mellitus, n (%)	30 (21.9%)	13 (17.8%)	0.603
Cardiovascular disease, n (%)	23 (16.8%)	8 (11.0%)	0.352
Aspirin use, n (%)	15 (10.9%)	11 (15.1%)	0.520
Laboratory and screening variables			
FIT (positive), n (%)	31 (22.6%)	20 (27.4%)	0.549
CRP, mg/L	3.28 (10.50)	3.91 (10.40)	0.678
CEA, ng/mL	2.25 (1.44)	2.53 (1.75)	0.238
CA19-9, U/mL	7.49 (8.48)	7.20 (6.71)	0.787
CA72-4, U/mL	4.03 (8.30)	2.01 (1.42)	0.006

Values are presented as mean (SD) or median (interquartile range), as appropriate, for continuous variables and number (percentage, %) for categorical variables. Comparisons between groups were performed using the Student’s t-test or Mann–Whitney U test for continuous variables and the χ² test or Fisher’s exact test for categorical variables, as appropriate. FDR, first-degree relative; HRA, high-risk adenoma; BMI, body mass index; FIT, fecal immunochemical test; CRP, C-reactive protein; CEA, carcinoembryonic antigen; CA19-9, carbohydrate antigen 19-9; CA72-4, carbohydrate antigen 72-4.

HRAs were detected in 73 FDRs (34.8%), including 40 (38.1%) in the proband HRA group and 33 (31.4%) in the control group. This relatively modest difference suggests that proband HRA status alone may have limited discriminatory value for identifying high-risk individuals among FDRs.

Colonoscopic findings and adenoma characteristics according to the HRA status of FDRs are summarized in **Supplementary Table 1**. As expected by definition, all individuals in the HRA group had at least one colorectal adenoma, and a substantially higher proportion presented with advanced features, including adenomas ≥1 cm and ≥3 adenomas. Notably, FDRs with HRA exhibited a markedly greater adenoma burden and more extensive colonic involvement compared with those without HRA. This was reflected by significantly higher proportions of multiple adenomas (≥2), as well as both proximal and distal colon involvement. In terms of anatomical distribution, adenomas in the HRA group were more frequently observed across all colonic segments, including the rectum, sigmoid colon, and proximal colon regions, indicating a more widespread and diffuse disease pattern. These findings indicate that FDRs with HRAs not only meet the criteria for advanced lesions but also exhibit a higher adenoma burden and more extensive colonic involvement compared with those without HRAs.

### Univariable analysis

Univariable logistic regression analyses are presented in **Table 2**. Alcohol consumption was significantly associated with higher odds of HRAs (OR = 4.30, 95% CI: 1.54–11.98, P = 0.005). In contrast, higher CA72–4 levels were associated with lower odds of HRAs (OR = 0.86, 95% CI: 0.75–0.98, P = 0.026). Other variables were not significantly associated with HRAs in univariable analyses (**Table 2**).

**Table 2:** Univariable logistic regression analysis of factors associated with HRA among FDRs.

Characteristic	Value	OR (95% CI)	P value
Demographics			
Age, years	61.9 (12.1)	1.02 (0.99–1.04)	0.173
Male sex, n (%)	106 (50.5%)	1.68 (0.95–2.99)	0.073
BMI, kg/m²	23.6 (3.4)	1.06 (0.97–1.15)	0.194
Lifestyle factors			
Smokers, n (%)	35 (16.7%)	1.51 (0.72–3.17)	0.273
Alcohol consumption, n (%)	18 (8.6%)	4.30 (1.54–11.98)	0.005
Medical history			
Proband HRA status, n (%)	105 (50.0%)	1.34 (0.76–2.37)	0.311
Hypertension, n (%)	97 (46.2%)	1.32 (0.75–2.33)	0.341
Diabetes mellitus, n (%)	43 (20.5%)	0.77 (0.37–1.59)	0.485
Cardiovascular disease, n (%)	31 (14.8%)	0.61 (0.26–1.44)	0.260
Aspirin use, n (%)	26 (12.4%)	1.44 (0.63–3.33)	0.390
Laboratory and screening variables			
FIT (positive), n (%)	51 (24.3%)	1.29 (0.67–2.48)	0.443
CRP, mg/L	3.5 (10.5)	1.01 (0.98–1.03)	0.680
CEA, ng/mL	2.3 (1.6)	1.12 (0.94–1.34)	0.217
CA19-9, U/mL	7.4 (7.9)	1.00 (0.96–1.03)	0.800
CA72-4, U/mL	3.3 (6.8)	0.86 (0.75–0.98)	0.026

Values are presented as mean (SD) or median (interquartile range), as appropriate, for continuous variables and number (percentage) for categorical variables. Odds ratios (ORs) and 95% confidence intervals (CIs) were estimated using univariable logistic regression models. A two-sided P value < 0.05 was considered statistically significant. BMI, body mass index; HRA, high-risk adenoma; CRP, C-reactive protein; CEA, carcinoembryonic antigen; CA19-9, carbohydrate antigen 19-9; CA72-4, carbohydrate antigen 72-4; FIT, fecal immunochemical test; OR, odds ratio; CI, confidence interval.

### Multivariable analysis

Multivariable logistic regression results for alcohol consumption are shown in **Table 3**. Alcohol consumption remained significantly associated with HRAs across all models. In the fully adjusted model (Model 3), alcohol consumption was associated with higher odds of HRAs (OR = 5.75, 95% CI: 1.49–22.20, P = 0.011), and this association remained consistent after progressive adjustment for potential confounders.

**Table 3:** Multivariable logistic regression analysis of the association between alcohol consumption and HRAs.

Characteristic	N (%)	Model 1	Model 2	Model 3
Alcohol consumption				
No	192 (91.4%)	Ref	Ref	Ref
Yes	18 (8.6%)	4.30 (1.54–11.98); P = 0.005	6.58 (1.82–23.81); P = 0.004	5.75 (1.49–22.20); P = 0.011

Model 1: unadjusted.

Model 2: adjusted for proband HRA status, age, sex, body mass index (BMI), and smoking status.

Model 3: further adjusted for aspirin use, hypertension, diabetes mellitus, cardiovascular disease, C-reactive protein (CRP), carcinoembryonic antigen (CEA), carbohydrate antigen 19-9 (CA19-9), carbohydrate antigen 72-4 (CA72-4), and fecal immunochemical test (FIT).

Values are presented as odds ratios (ORs) with 95% confidence intervals (CIs). P values were calculated from logistic regression models. A two-sided P value < 0.05 was considered statistically significant.

In contrast, proband HRA status was not significantly associated with HRA risk in FDRs after adjustment, suggesting limited incremental value for risk stratification beyond individual-level factors. For CA72-4, the inverse association observed in univariable and partially adjusted models was attenuated after full adjustment, and no clear dose–response relationship was observed across quartiles (**Supplementary Table 2**). These findings suggest that the initial association may be influenced by confounding factors rather than representing an independent effect.

### Bootstrap validation

To further assess the robustness of the association between alcohol consumption and HRAs, bootstrap resampling (5,000 iterations) was performed based on the fully adjusted model (Model 3).

In the original multivariable analysis, alcohol consumption was significantly associated with higher odds of HRAs (OR = 5.75, 95% CI: 1.49–22.20, P = 0.011). Bootstrap analysis yielded a median OR of 7.37 (bootstrap 95% CI: 1.53–63.63). Across the resampled datasets, 99.38% of OR estimates were greater than 1, and 74.28% of P values were < 0.05.

### Model performance

**Figure 1:**
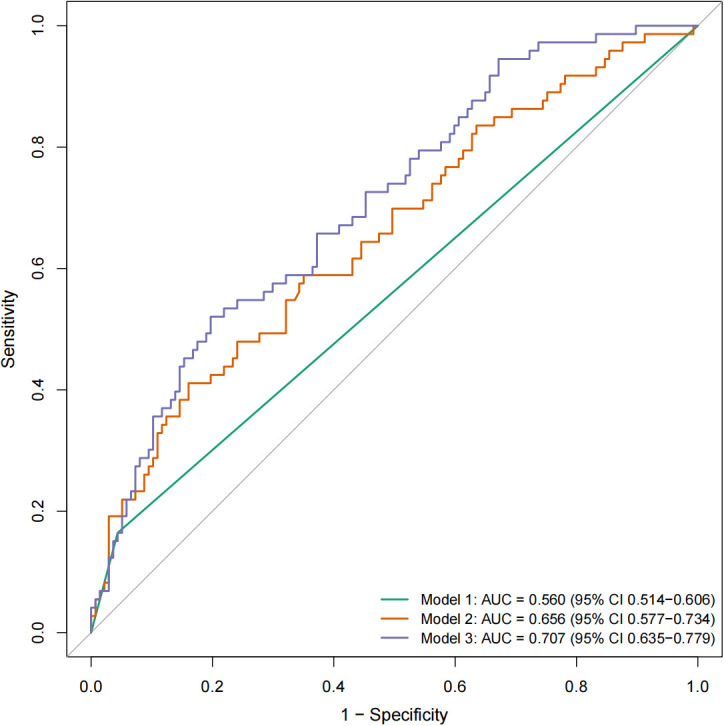
Receiver operating characteristic (ROC) curves of three logistic regression models for predicting HRAs. The ROC curves illustrate the discriminative performance of three logistic regression models for predicting HRAs. Model 1 was an unadjusted model including alcohol consumption as the sole predictor. Model 2 was adjusted for proband HRA status, age, sex, body mass index, and smoking status. Model 3 was further adjusted for clinical variables (aspirin use, hypertension, diabetes mellitus, and cardiovascular disease), inflammatory markers (C-reactive protein), tumor biomarkers (CEA, CA19-9, and CA72-4), and fecal immunochemical testing (FIT). The AUC increased from 0.560 (95% CI 0.514–0.606) in Model 1 to 0.656 (95% CI 0.577–0.734) in Model 2 and 0.707 (95% CI 0.635–0.779) in Model 3, indicating improved discrimination with incremental adjustment. The diagonal reference line represents a non-informative model (AUC = 0.5).

The performance of the fully adjusted model (Model 3) was comprehensively evaluated in terms of discrimination, calibration, and clinical utility. The model demonstrated moderate discriminative ability, with an AUC of 0.707 (**Figure 1**). Calibration analysis showed good agreement between predicted and observed probabilities, with the bias-corrected calibration curve closely approximating the ideal reference line and a mean absolute error of 0.052 (**Supplementary Figure 2**). DCA indicated that the model provided a higher net benefit than both the treat-all and treat-none strategies across low-to-intermediate threshold probabilities, particularly between 0.00 and 0.66. However, the net benefit became less stable at higher thresholds, suggesting that the clinical utility of the model may be limited in decision-making scenarios requiring higher risk thresholds (**Supplementary Figure 3**).

### Subgroup and interaction analyses

**Figure 2:**
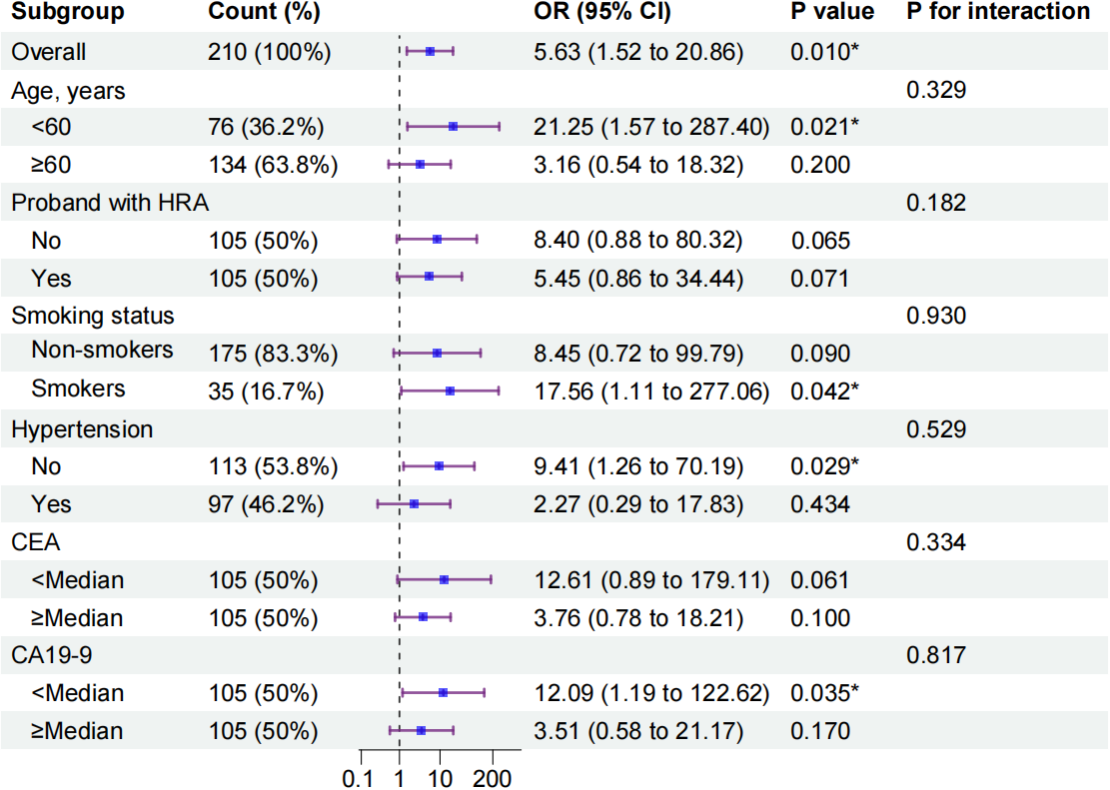
Subgroup analysis of the association between alcohol consumption and HRAs among first-degree relatives. P values represent the statistical significance of the association within each subgroup based on logistic regression models, and P for interaction indicates statistical evidence of effect modification across subgroups. Due to sparse data and insufficient event counts in certain strata, variables including aspirin use, diabetes mellitus, cardiovascular disease, and sex were excluded from subgroup analyses to avoid unstable estimates. Asterisks indicate subgroup-specific P values < 0.05. The vertical dashed line at OR = 1 indicates no association.

In stratified analyses, alcohol consumption showed a consistent positive association with HRAs across several subgroups, including participants aged <60 years, smokers, individuals without hypertension, and those with lower CA19–9 levels. However, no statistically significant interactions were observed (P for interaction > 0.05), indicating that the magnitude of association did not differ significantly across strata (**Figure 2**). Given the limited sample size and multiple comparisons, these subgroup analyses should be considered exploratory and interpreted with caution. The findings require validation in larger, independent cohorts.

## Discussion

In this observational case–control study of first-degree relatives (FDRs) of colonoscopy-evaluated probands, HRAs were detected in 34.8% of FDRs in the study sample, indicating a substantial burden of advanced precancerous lesions in this population. Alcohol consumption emerged as an important and potentially modifiable independent factor associated with HRAs, whereas proband HRA status was not significantly associated with HRAs among FDRs. In this context, the predictive model may be useful in identifying FDRs who could benefit from earlier or more frequent colonoscopic screening, particularly within low-to-intermediate risk thresholds where the model demonstrated the most stable net benefit. Notably, the relatively modest difference in HRA prevalence between groups compared with the originally assumed effect size may have reduced statistical power to detect associations with variables of smaller effect sizes.

### Substantial detection of HRAs among FDRs

In this selected case–control study sample, HRAs were detected in 34.8% of FDRs. Although this proportion should not be interpreted as a population prevalence estimate, it was higher than the proportions reported in average-risk screening populations. A systematic review and meta-analysis in average-risk individuals estimated a pooled advanced adenoma prevalence of 5.7%, with variation largely driven by age distribution (21). In routine clinical practice, advanced adenomas are typically detected in approximately 10% of individuals undergoing screening colonoscopy, further highlighting the substantial burden observed in our study sample (22).

This finding is consistent with prior evidence demonstrating familial aggregation of colorectal neoplasia. Colonoscopy-based studies have shown that first-degree relatives (FDRs) of individuals with advanced adenomas have an increased risk of colorectal tumors (8). In addition, a recent meta-analysis reported that family history of colorectal cancer in FDRs is associated with an increased risk of adenoma and advanced neoplasia, although effect sizes vary across populations and study designs (23).

From a clinical perspective, the substantial proportion of HRAs detected in this study underscores the substantial burden of advanced precancerous lesions in FDRs and supports the need for targeted screening strategies in this population. These findings are aligned with current guideline recommendations, which advocate earlier and colonoscopy-based screening for individuals with a first-degree relative affected by colorectal cancer or advanced adenomas (4, 24). In the present study, FDRs with HRA not only met the diagnostic criteria for advanced adenomas but also exhibited a substantially greater adenoma burden and more extensive colonic involvement compared with those without HRA. Importantly, these differences extended beyond the defining features of HRA, as individuals in the HRA group showed a higher proportion of multiple adenomas and involvement of both proximal and distal colon segments.

This pattern suggests a more diffuse distribution of adenomas among FDRs with HRA, rather than a localized lesion process. Such widespread distribution across colonic segments may reflect an underlying field effect or shared susceptibility along the colorectal mucosa. This diffuse distribution may be partially explained by shared genetic susceptibility or environmental exposures within families, although further studies are needed to clarify the underlying mechanisms. From a clinical perspective, these findings underscore the importance of comprehensive colonoscopic evaluation in FDRs at higher risk, as limited or segmental screening approaches may fail to detect clinically relevant lesions. The observed distribution pattern further supports the use of full colonoscopy rather than stool-based screening modalities in this population.

### Alcohol consumption as a key modifiable risk factor

Alcohol consumption emerged as an important independent factor associated with HRAs in this study. This finding is consistent with epidemiological evidence linking alcohol intake to colorectal neoplasia. A meta-analysis focusing on colorectal adenomas demonstrated that alcohol consumption is associated with increased adenoma risk, with evidence of dose–response relationships in several populations (25). Similarly, at the level of colorectal cancer, a large dose–response meta-analysis has shown a progressive increase in risk with higher alcohol intake (26).

Although alcohol exposure in our study was assessed as a binary variable without detailed quantification, this limitation may have led to exposure misclassification and prevented assessment of potential dose–response relationships, which could influence the magnitude and interpretation of the observed association.

Alcohol metabolism produces acetaldehyde, a genotoxic metabolite. This compound induces DNA damage and oxidative stress and affects folate metabolism, epigenetic regulation, and mucosal integrity. These mechanisms, together with effects on gut microbiota and inflammation, are thought to contribute to colorectal carcinogenesis (27–30).

In the context of FDRs, alcohol may further interact with underlying genetic susceptibility, potentially accelerating adenoma development in high-risk individuals. Although genetic data were not available in this study, emerging evidence suggests that polygenic risk may modify the effects of lifestyle exposures, highlighting a potential gene–environment interaction framework (31).

From a clinical perspective, alcohol represents a modifiable risk factor with direct implications for prevention. Incorporating alcohol history into risk assessment may help identify higher-risk individuals, while targeted counseling could complement colonoscopy-based screening strategies in this population.

### Lack of independent association of proband HRA status

In the present study, proband HRA status was not significantly associated with HRA risk in FDRs in univariable analysis (P > 0.20). Therefore, the anticipated association between proband HRA status and HRA detection among FDRs was not observed. Although the point estimate suggested a higher risk, the association did not reach statistical significance. This result suggests that proband HRA status alone may have limited discriminatory value for risk stratification within FDR populations.

One possible explanation is that family history represents a heterogeneous construct encompassing both genetic susceptibility and shared environmental exposures. Such heterogeneity may attenuate the predictive value of proband status when considered in isolation. Consistent with this interpretation, prior studies have reported an overall increased risk of colorectal neoplasia among FDRs, but with substantial variability in effect sizes across populations and outcome definitions (23). These findings highlight the need for more refined risk stratification approaches beyond simple family history.

This observation may be partly explained by limited statistical power. The observed difference in the proportion of FDRs with HRAs between groups (38.1% vs. 31.4%) was smaller than the effect size assumed in the sample size calculation (50% vs. 30%), which may have reduced the ability to detect modest associations for certain variables, including biomarkers such as CA72-4.

### Limited value of FIT and CA72-4

In this study, FIT was not independently associated with HRAs, and its contribution to risk stratification was limited. This is consistent with prior evidence demonstrating that FIT has relatively low sensitivity for advanced adenomas, even though its performance for colorectal cancer detection is comparatively higher. A systematic review and meta-analysis reported a sensitivity of approximately 0.40 for advanced adenomas at a commonly used threshold of 10 µg/g (12). Similarly, pooled analyses have shown suboptimal performance of FIT in detecting colorectal polyps, reinforcing its limitations for identifying precursor lesions (32).

These limitations are biologically plausible, as many adenomas bleed intermittently or minimally, reducing the likelihood of detection by stool-based testing. In addition, FIT thresholds in screening programs are typically optimized for colorectal cancer detection rather than for identifying advanced adenomas.

Consistent with these considerations, our findings support colonoscopy as the preferred screening modality for FDRs when the primary goal is prevention through detection and removal of advanced precursor lesions. FIT may still serve as a pragmatic alternative in settings where colonoscopy is declined or not readily available, in line with current guideline recommendations (4, 8).

Regarding CA72-4, although an inverse association with HRAs was observed in univariable analysis, this finding was not consistent in multivariable models. These findings suggest that the apparent association observed in univariable analysis was likely influenced by confounding factors, and CA72–4 cannot be considered an independent predictive biomarker for HRAs.

### Model performance and clinical implications

The fully adjusted model (Model 3) demonstrated moderate discriminative ability (AUC = 0.707), indicating modest performance for individual-level prediction. However, DCA suggested that the model may provide net clinical benefit across low-to-intermediate threshold probabilities, supporting its potential utility for risk stratification. Clinically, this suggests that the model may be most useful in identifying FDRs who could benefit from intensified colonoscopic surveillance, particularly in low-to-intermediate risk scenarios, whereas its utility at higher thresholds appears limited due to less stable net benefit. This may be particularly relevant in clinical settings where decisions regarding screening intensity need to be individualized based on estimated risk.

Importantly, bootstrap resampling provided internal validation of the model and demonstrated stable effect estimates, partially mitigating concerns regarding model overfitting. Nevertheless, the predictive performance remains modest, suggesting that key determinants of HRA risk may not be fully captured by routinely available clinical and laboratory variables.

Future research should focus on integrating additional predictors, including genetic susceptibility (e.g., polygenic risk scores), more detailed lifestyle exposures, and emerging biomarkers. Moreover, validation in larger and more diverse populations is needed to improve model robustness, generalizability, and overall predictive performance.

### Strengths and limitations

This study has several strengths. First, all included FDRs underwent colonoscopy and histopathological assessment, which ensured accurate and uniform ascertainment of HRA status. Second, FDRs were enrolled from two proband-defined recruitment groups, and frequency matching by age and sex improved comparability between these groups; moreover, age and sex were further adjusted for in the association analyses. Third, baseline demographic, lifestyle, clinical, laboratory, FIT, and colonoscopy-related variables were collected using standardized procedures, which helped minimize measurement variability. Fourth, bootstrap resampling provided internal validation and supported the robustness of the main association between alcohol consumption and HRAs. Finally, the exploratory prediction model was evaluated using multiple complementary metrics, including discrimination, calibration, and decision curve analysis, allowing a multidimensional assessment of model performance and potential clinical utility.

Several limitations should be acknowledged. First, this was a single-center study conducted in a relatively homogeneous population, which may limit generalizability to other populations. Regional genetic and environmental factors may have influenced the observed associations. Second, the sample size was moderate, particularly for subgroup analyses, resulting in wide confidence intervals. Therefore, subgroup findings should be considered exploratory and interpreted with caution. Third, alcohol consumption was self-reported and assessed as a binary variable without information on dose, duration, or type of alcohol intake, precluding evaluation of dose–response relationships, which may affect the interpretation of its role in HRA development and may also lead to exposure misclassification. Fourth, key lifestyle and behavioral factors, including dietary patterns (e.g., red meat and fiber intake) and physical activity, were not available and may have introduced residual confounding. Although aspirin use was included as a covariate, information on other nonsteroidal anti-inflammatory drugs (NSAIDs) was not available, which may have resulted in incomplete adjustment for this class of medications. Finally, although internal validation was performed using bootstrap resampling, external validation was not conducted. Therefore, the generalizability and clinical applicability of the model require further confirmation in independent study populations.

### Conclusions

In conclusion, HRAs were detected in a substantial proportion of first-degree relatives in this observational case–control study, highlighting the importance of colonoscopy-based screening in this population. Alcohol consumption was identified as a modifiable factor associated with HRAs, whereas proband HRA status and FIT provided limited additional value for risk stratification. These findings support the integration of lifestyle factors, particularly alcohol consumption, into risk assessment strategies for FDRs. Although the predictive model demonstrated moderate discriminative ability, it may offer some clinical utility in risk stratification. These findings support the prioritization of colonoscopy-based screening and targeted lifestyle counseling, particularly alcohol-related counseling, among colonoscopy-evaluated FDRs regardless of proband HRA status. Future studies incorporating genetic and environmental factors are needed to develop more precise risk stratification strategies.

## Data Availability

The datasets generated and analyzed during the current study are available from the corresponding author upon reasonable request, subject to ethical and privacy restrictions.

## References

[1] Bray F Laversanne M Sung H Ferlay J Siegel RL Soerjomataram I . Global cancer statistics 2022: GLOBOCAN estimates of incidence and mortality worldwide for 36 cancers in 185 countries. CA Cancer J Clin. (2024) 74:229–63. doi: 10.3322/caac.21834. PMID: 38572751

[2] Winawer SJ Zauber AG Ho MN O’Brien MJ Gottlieb LS Sternberg SS . Prevention of colorectal cancer by colonoscopic polypectomy. The National Polyp Study Workgroup. N Engl J Med. (1993) 329:1977–81. doi: 10.1056/NEJM199312303292701. PMID: 8247072

[3] Zauber AG Winawer SJ O’Brien MJ Lansdorp-Vogelaar I van Ballegooijen M Hankey BF . Colonoscopic polypectomy and long-term prevention of colorectal-cancer deaths. N Engl J Med. (2012) 366:687–96. doi: 10.1056/NEJMoa1100370. PMID: 22356322 PMC3322371

[4] Shaukat A Kahi CJ Burke CA Rabeneck L Sauer BG Rex DK . ACG Clinical Guidelines: Colorectal Cancer Screening 2021. Am J Gastroenterol. (2021) 116:458–79. doi: 10.14309/ajg.0000000000001122. PMID: 33657038

[5] Davidson KW Barry MJ Mangione CM Cabana M Caughey AB Davis EM . Screening for Colorectal Cancer: US Preventive Services Task Force Recommendation Statement. JAMA. (2021) 325:1965–77. doi: 10.1001/jama.2021.6238. PMID: 34003218

[6] Rex DK Boland CR Dominitz JA Giardiello FM Johnson DA Kaltenbach T . Colorectal cancer screening: Recommendations for physicians and patients from the U.S. Multi-Society Task Force on Colorectal Cancer. Gastrointest Endosc. (2017) 86:18–33. doi: 10.1016/j.gie.2017.04.003. PMID: 28600070

[7] Wong MCS Ching JYL Chiu H-M Wu KC Rerknimitr R Li J . Risk of colorectal neoplasia in individuals with self-reported family history: A prospective colonoscopy study from 16 Asia-Pacific regions. Am J Gastroenterol. (2016) 111:1621–9. doi: 10.1038/ajg.2016.52. PMID: 26977757

[8] Cottet V Pariente A Nalet B Lafon J Milan C Olschwang S . Colonoscopic screening of first-degree relatives of patients with large adenomas: increased risk of colorectal tumors. Gastroenterology. (2007) 133:1086–92. doi: 10.1053/j.gastro.2007.07.023. PMID: 17919484

[9] Ng SC Lau JYW Chan FKL Suen BY Tse YK Hui AJ . Risk of advanced adenomas in siblings of individuals with advanced adenomas: A cross-sectional study. Gastroenterology. (2016) 150:608–16. doi: 10.1053/j.gastro.2015.11.003. PMID: 26584600

[10] Lee T Teng TZJ Shelat VG . Carbohydrate antigen 19-9 - tumor marker: Past, present, and future. World J Gastrointest Surg. (2020) 12:468–90. doi: 10.4240/wjgs.v12.i12.468. PMID: 33437400 PMC7769746

[11] Xu Y Zhang P Zhang K Huang C . The application of CA72–4 in the diagnosis, prognosis, and treatment of gastric cancer. Biochim Biophys Acta Rev Cancer. (2021) 1876:188634. doi: 10.1016/j.bbcan.2021.188634. PMID: 34656687

[12] Imperiale TF Gruber RN Stump TE Emmett TW Monahan PO . Performance characteristics of fecal immunochemical tests for colorectal cancer and advanced adenomatous polyps: A systematic review and meta-analysis. Ann Intern Med. (2019) 170:319–29. doi: 10.7326/M18-2390. PMID: 30802902

[13] Wong MCS Huang J Huang JLW Pang TWY Choi P Wang J . Global prevalence of colorectal neoplasia: A systematic review and meta-analysis. Clin Gastroenterol Hepatol. (2020) 18:553–61.e10. doi: 10.1016/j.cgh.2019.07.016. PMID: 31323383

[14] Chiu H-M Lee Y-C Tu C-H Chen C-C Tseng P-H Liang J-T . Association between early stage colon neoplasms and false-negative results from the fecal immunochemical test. Clin Gastroenterol Hepatol. (2013) 11:832–8.e1–2. doi: 10.1016/j.cgh.2013.01.013. PMID: 23376002

[15] Keum N Giovannucci E . Global burden of colorectal cancer: emerging trends, risk factors and prevention strategies. Nat Rev Gastroenterol Hepatol. (2019) 16:713–32. doi: 10.1038/s41575-019-0189-8. PMID: 31455888

[16] Robertson DJ Lee JK Boland CR Dominitz JA Giardiello FM Johnson DA . Recommendations on fecal immunochemical testing to screen for colorectal neoplasia: A consensus statement by the US Multi-Society Task Force on Colorectal Cancer. Gastroenterology. (2017) 152:1217–37.e3. doi: 10.1053/j.gastro.2016.08.053. PMID: 27769517

[17] Clark BT Protiva P Nagar A Imaeda A Ciarleglio MM Deng Y . Quantification of adequate bowel preparation for screening or surveillance colonoscopy in men. Gastroenterology. (2016) 150:396–405. doi: 10.1053/j.gastro.2015.09.041. PMID: 26439436 PMC4728019

[18] Nagtegaal ID Odze RD Klimstra D Paradis V Rugge M Schirmacher P . The 2019 WHO classification of tumours of the digestive system. Histopathology. (2020) 76:182–8. doi: 10.1111/his.13975. PMID: 31433515 PMC7003895

[19] Hassan C Antonelli G Dumonceau J-M Regula J Bretthauer M Chaussade S . Post-polypectomy colonoscopy surveillance: European Society of Gastrointestinal Endoscopy (ESGE) Guideline - Update 2020. Endoscopy. (2020) 52:687–700. doi: 10.1055/a-1185-3109. PMID: 32572858

[20] Gupta S Lieberman D Anderson JC Burke CA Dominitz JA Kaltenbach T . Recommendations for follow-up after colonoscopy and polypectomy: A consensus update by the US Multi-Society Task Force on Colorectal Cancer. Gastroenterology. (2020) 158:1131–53.e5. doi: 10.1053/j.gastro.2019.10.026. PMID: 32044092 PMC7672705

[21] Heitman SJ Ronksley PE Hilsden RJ Manns BJ Rostom A Hemmelgarn BR . Prevalence of adenomas and colorectal cancer in average risk individuals: a systematic review and meta-analysis. Clin Gastroenterol Hepatol. (2009) 7:1272–8. doi: 10.1016/j.cgh.2009.05.032. PMID: 19523536

[22] Molmenti CL Kolb JM Karlitz JJ . Advanced colorectal polyps on colonoscopy: A trigger for earlier screening of family members. Am J Gastroenterol. (2020) 115:311–4. doi: 10.14309/ajg.0000000000000467. PMID: 31977326 PMC7094814

[23] Gao K Jin H Yang Y Li J He Y Zhou R . Family history of colorectal cancer and the risk of colorectal neoplasia: A systematic review and meta-analysis. Am J Gastroenterol. (2025) 120:531–9. doi: 10.14309/ajg.0000000000003120. PMID: 39513348

[24] Wilkinson AN Lieberman D Leontiadis GI Tse F Barkun AN Abou-Setta A . Colorectal cancer screening for patients with a family history of colorectal cancer or adenomas. Can Fam Physician. (2019) 65:784–9. doi: 10.1201/9781315385310-4 PMC6853346 31722908

[25] Zhu J-Z Wang Y-M Zhou Q-Y Zhu K-F Yu C-H Li Y-M . Systematic review with meta-analysis: alcohol consumption and the risk of colorectal adenoma. Aliment Pharmacol Ther. (2014) 40:325–37. doi: 10.1111/apt.12841. PMID: 24943329

[26] Fedirko V Tramacere I Bagnardi V Rota M Scotti L Islami F . Alcohol drinking and colorectal cancer risk: an overall and dose-response meta-analysis of published studies. Ann Oncol. (2011) 22:1958–72. doi: 10.1093/annonc/mdq653. PMID: 21307158

[27] Kim Y-I . Role of folate in colon cancer development and progression. J Nutr. (2003) 133:3731S–9S. doi: 10.1093/jn/133.11.3731S. PMID: 14608107

[28] Johnson CH Golla JP Dioletis E Singh S Ishii M Charkoftaki G . Molecular mechanisms of alcohol-induced colorectal carcinogenesis. Cancers (Basel). (2021) 13:4404. doi: 10.3390/cancers13174404. PMID: 34503214 PMC8431530

[29] Rumgay H Murphy N Ferrari P Soerjomataram I . Alcohol and cancer: Epidemiology and biological mechanisms. Nutrients. (2021) 13:3173. doi: 10.3390/nu13093173. PMID: 34579050 PMC8470184

[30] Seitz HK . A narrative review on alcohol and alimentary tract cancer with special emphasis on acetaldehyde and oxidative stress. Z Gastroenterol. (2025) 63:960–74. doi: 10.1055/a-2588-6849. PMID: 40378880

[31] Tamlander M Jermy B Seppälä TT Färkkilä M Widén E Ripatti S . Genome-wide polygenic risk scores for colorectal cancer have implications for risk-based screening. Br J Cancer. (2024) 130:651–9. doi: 10.1038/s41416-023-02536-z. PMID: 38172535 PMC10876651

[32] Afzal A Aranan YS Roberts T Covington J Vidal L Ahmed S . Diagnostic accuracy of the faecal immunochemical test and volatile organic compound analysis in detecting colorectal polyps: meta-analysis. BJS Open. (2024) 9:zrae154. doi: 10.1093/bjsopen/zrae154. PMID: 39972538 PMC11839406

