# Supplementary material for: Colonoscopy-detected high-risk adenomas and their association with alcohol consumption among first-degree relatives: an observational case–control study
Source: Front Oncol. 2026 May 21;16:1791247. doi: 10.3389/fonc.2026.1791247 (PMC13234566; doi:10.3389/fonc.2026.1791247)

Supplementary Table S1. Colonoscopic findings and adenoma characteristics of first-degree relatives according to their own HRA status.

Characteristic	FDRs without HRA (N = 137)	FDRs with HRA (N = 73)
**Adenoma status**		
Any colorectal adenoma	16 (11.7%)	73 (100%)
Adenoma ≥ 1 cm	0 (0.0%)	57 (78.1%)
≥ 3 adenomas	1 (0.7%)	47 (64.4%)
Proximal colon involvement	11 (8.0%)	53 (72.6%)
Distal colon involvement	8 (5.8%)	53 (72.6%)
Multiple adenomas (≥2)	5 (3.7%)	54 (74.0%)
**Adenoma location**		
Rectum	5 (3.7%)	18 (24.7%)
Sigmoid colon	10 (7.3%)	27 (37.0%)
Descending colon	2 (1.5%)	22 (30.1%)
Transverse colon	6 (4.4%)	31 (42.5%)
Hepatic flexure	3 (2.2%)	11 (15.1%)
Ascending colon	6 (4.4%)	33 (45.2%)
Ileocecal region	1 (0.7%)	8 (11.0%)

Values are presented as number (percentage, %). Percentages for adenoma location are not mutually exclusive and were calculated using all participants within each FDR HRA-status group as the denominator. As expected by definition, all individuals in the HRA group had at least one adenoma. Variables inherent to the definition of HRA are presented descriptively, and no formal P values are shown.

Abbreviations: FDR, first-degree relative; HRA, high-risk adenoma.

Supplementary Table S2. Association between serum CA72-4 levels and odds of high-risk adenoma detection in multivariable logistic regression analyses.

Characteristic	Statistics	Model 1	Model 2	Model 3
**CA72-4**				
**Per SD increase**		0.35 (0.14 – 0.88); P = 0.026	0.35 (0.13 – 0.97); P = 0.043	0.39 (0.14 – 1.08); P = 0.069
Q1	53 (25.24%)	Ref	Ref	Ref
Q2	52 (24.76%)	3.19 (1.42 – 7.18); P = 0.005	3.01 (1.30 – 6.98); P = 0.010	3.63 (1.47 – 8.99); P = 0.005
Q3	52 (24.76%)	1.23 (0.54 – 2.83); P = 0.625	1.21 (0.52 – 2.84); P = 0.660	1.39 (0.57 – 3.39); P = 0.473
Q4	53 (25.24%)	0.74 (0.31 – 1.78); P = 0.504	0.77 (0.31 – 1.91); P = 0.568	0.88 (0.34 – 2.29); P = 0.796
P for trend		0.176	0.264	0.427

Abbreviations: CA72-4, carbohydrate antigen 72-4; SD, standard deviation; OR, odds ratio; CI, confidence interval; BMI, body mass index; CRP, C-reactive protein; FIT, fecal immunochemical test.

Model 1 was unadjusted.

Model 2 was adjusted for proband HRA status, age, sex, body mass index (BMI), and smoking status.

Model 3 was further adjusted for aspirin use, hypertension, diabetes mellitus, cardiovascular disease, C-reactive protein (CRP), alcohol consumption, and FIT.

CA72-4 was analyzed both as a continuous variable (per SD increase) and as a categorical variable based on quartiles (Q1–Q4), with Q1 as the reference group.

P for trend was calculated by assigning the median value of each quartile and modeling it as a continuous variable. CEA and CA19-9 were not included in this model to avoid collinearity.

Analyses were conducted using the imputed dataset generated by the missForest algorithm to account for missing data.

Supplementary Figure S1. Study flowchart and participant selection.
Among 267 initially assessed first-degree relatives (FDRs) who underwent colonoscopy screening, 57 participants were excluded because of incomplete colonoscopy or inadequate bowel preparation (n = 36), missing key variables (n = 8), or a history of colorectal cancer or other major colorectal diseases (n = 13). A total of 210 FDRs were included in the final analysis.

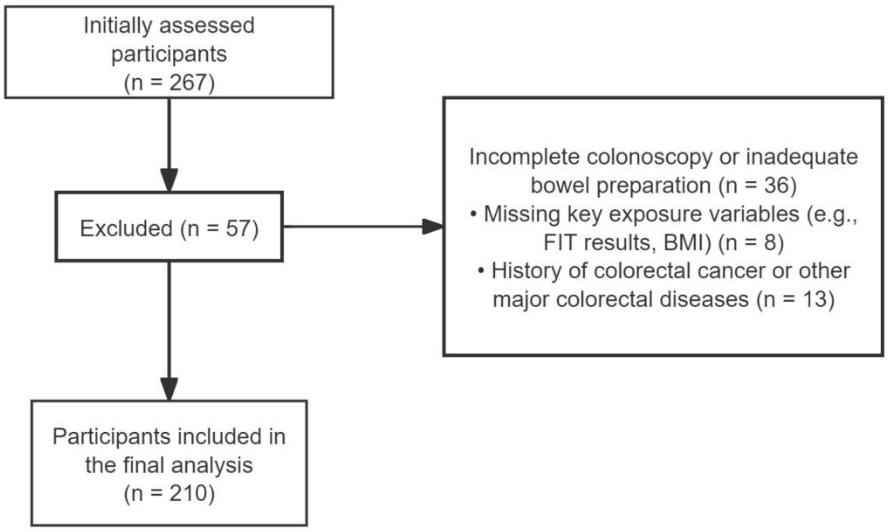

Supplementary Figure S2. Calibration curve of Model 3 for predicting HRA.

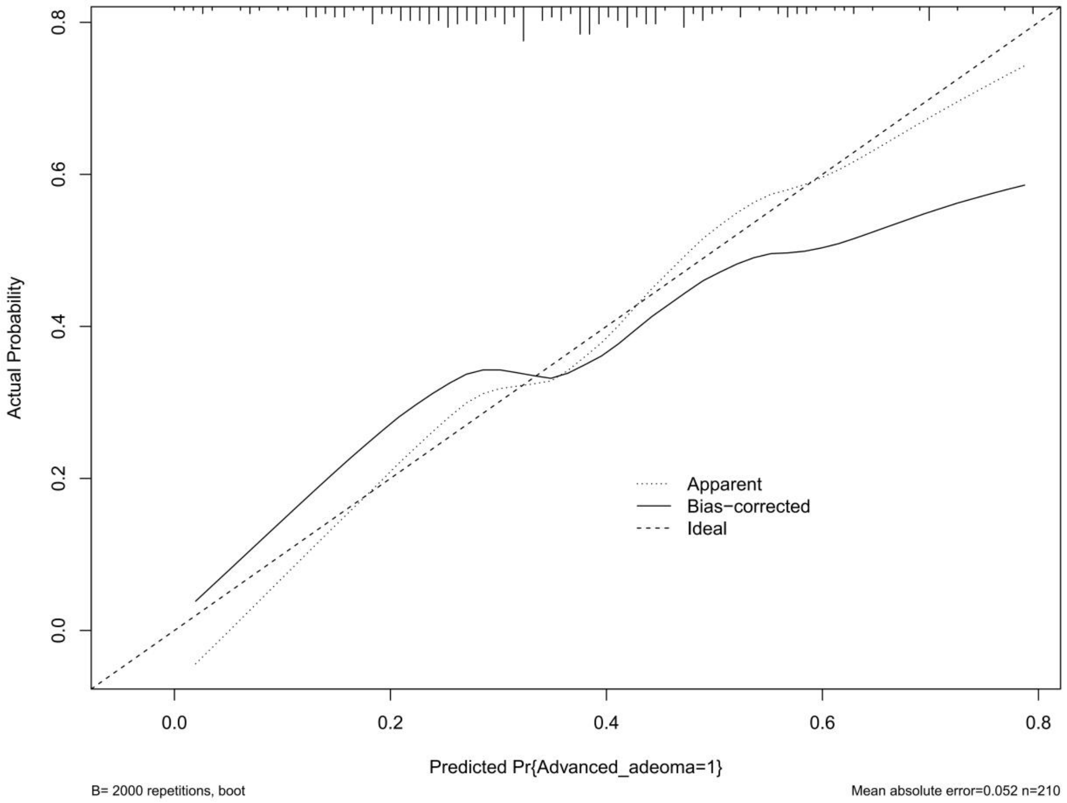

The calibration curve evaluates the agreement between predicted and observed probabilities. The dashed diagonal line indicates perfect calibration. The dotted line represents the apparent model performance, and the solid line represents the bias-corrected performance obtained using bootstrap resampling (B = 2000). The model demonstrated acceptable calibration with good agreement between predicted and observed probabilities (mean absolute error = 0.052).

Supplementary Figure S3. Decision curve analysis (DCA) of Model 3 for predicting HRA.

DCA was performed to evaluate the clinical utility of Model 3. The x-axis represents the threshold probability, and the y-axis represents the net benefit. Model 3 provided a higher net benefit than both "treat-all" and "treat-none" strategies across multiple threshold ranges, suggesting potential clinical usefulness, particularly within low-to-intermediate threshold probabilities.

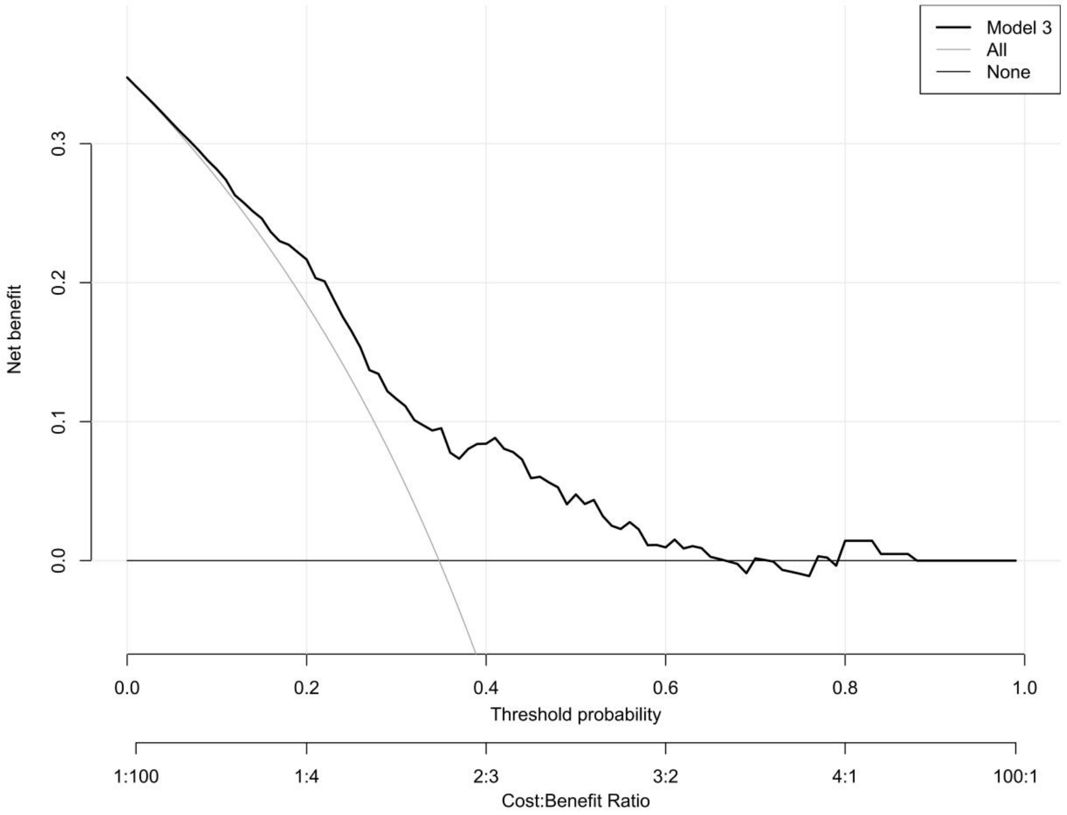

Supplement: Supplementary file 1 [file DataSheet1.pdf]
